# Educational background of professionals managing health services in Brazil: insights from the national registry of specialization and certification course offerings

**DOI:** 10.3389/fmed.2025.1695937

**Published:** 2026-01-08

**Authors:** Ericles Andrei Bellei, Debora Cristina Bellei, Cleide Fátima Moretto, Ana Luisa Sant' Anna Alves, Ana Carolina Bertoletti De Marchi

**Affiliations:** 1 Institute of Health, University of Passo Fundo (UPF), Passo Fundo, RS, Brazil; 2 School of Medicine, Community University of Chapecó Region (Unochapecó), Chapecó, SC, Brazil; 3 School of Agricultural Sciences, Innovation and Business, University of Passo Fundo (UPF), Passo Fundo, RS, Brazil; 4 Institute of Technology, University of Passo Fundo (UPF), Passo Fundo, RS, Brazil

**Keywords:** Brazilian national health system, case managers, curriculum and instruction, health care facilities workforce and services, health services, health services administration, interprofessional education, public health professional education

## 1 Introduction

Health services management differs from the traditional definition of a profession, as it lacks a standardized body of knowledge and a single entry credential (1). Instead, it is an applied field that integrates competencies from medicine, economics, public administration, engineering, and social sciences (2). Due to the need for diverse skill sets, no universal educational pathway exists for health service managers (3). Comparative studies demonstrate significant variation in managers' educational backgrounds, ranging from postgraduate qualifications to clinical experience without formal management training (4–6). Despite this diversity, the strategic importance of this workforce is increasing. Factors such as rising costs, demographic shifts, and heightened accountability expectations (7, 8) demand leaders capable of coordinating resources, interpreting data, and guiding improvement initiatives. The lack of a standardized pathway complicates the definition of professional training, underscoring the need to examine current educational preparation.

In Brazil, where a universal public health system operates alongside a substantial private sector and governance is decentralized, managerial preparation significantly influences access to care, operational efficiency, and the sustainability of services (9). While the education of physicians and other healthcare professionals has received increasing attention due to its impact on the system's future (10–12), the preparation of healthcare managers also merits close examination, as they are essential in primary care, hospitals, diagnostic networks, and regulatory agencies (13, 14). Job titles such as coordinator, supervisor, director, and manager encompass a range of functions that vary by setting and region (15). Professional competencies are developed through formal education, training, and ongoing professional development, with requirements differing by sector and management level (16). Delivering health services with consistent quality depends on the training and qualifications of those in leadership roles (17). In this context, a narrow professional definition is insufficient, as no universally mandated educational pathway exists.

Analyzing training and education offers a concrete approach to defining and understanding health service management as a profession. Systematic evaluation of courses can identify areas for improvement, inform decision-making, and clarify which topics are most relevant for graduates' societal roles (18). In the Brazilian higher education system, post-bachelor's programs are divided into two legal categories (19): *lato sensu* programs, which include specialization courses and MBAs classified as continuing education (20, 21), and *stricto sensu* programs, which are research-oriented master's and doctoral degrees. *Lato sensu* programs, offered by accredited institutions, require a minimum of 360 hours, emphasize practical skill development, and primarily serve professionals seeking additional credentials. In contrast, *stricto sensu* programs confer academic titles and develop research expertise.

Specialization programs play a critical role in professional upskilling and lifelong learning, but they face challenges as knowledge requirements shift in response to market and societal changes (18). Variability in duration, workload, delivery mode, and competency focus creates uncertainty regarding the knowledge and skills that graduates bring to the health system. *Lato sensu* courses are especially significant, with approximately 173,000 active offerings enrolling around 1.4 million students in 2023 (22). A flexible regulatory environment enables institutions to rapidly introduce or update programs, adapting curricula to emerging areas such as hospital quality improvement, digital health operations, and value-based care (21). These certificates, typically delivered in modular weekend, evening, or distance-learning formats, are accessible to professionals who continue working full-time. Consequently, analyzing these programs provides a practical means to map the educational pipelines that supply Brazil's managerial workforce.

Despite its significance, comprehensive evidence on national management curricula is limited, as most studies address only specific regions or small samples (15, 23). This lack of system-wide perspective hinders efforts to understand and improve training pathways. The Brazilian government maintains an official registry of specialization and certification programs, which, if analyzed, can clarify the educational content that defines the field on a national scale. Previous studies have not fully utilized this registry to provide a structured curriculum overview. This study addresses this gap by compiling a dataset of instructional topics from registered *lato sensu* specialization and certification courses in health services management. Rather than ranking programs or evaluating outcomes, it offers a descriptive, system-level reference to inform accreditation, workforce planning, employer expectations, and future educational assessments. By consolidating dispersed national data, this study establishes a foundation for targeted improvements and informed discussion among educators, employers, and regulators regarding the enhancement of management education.

## 2 Methods

### 2.1 Data acquisition

Initial data were extracted from the National Registry of Higher Education Courses and Institutions (24) (e-MEC, *Cadastro Nacional de Cursos e Instituições de Educação Superior*). This official electronic information system underpins the federal regulation, accreditation, and supervision of Brazilian higher-education institutions and their academic programs. The e-MEC platform began operations in January 2007. Subsequent normative acts, most recently a Ministerial Ordinance in 2017 (25), reaffirmed e-MEC as the exclusive environment for registering, updating, and disseminating course-level information within the federal higher education system. Since accreditation or major curricular changes require a new e-MEC submission, the registry provides a near-census view of all recognized courses since the mid-2000s.

Due to limitations in the e-MEC platform's search engine, we first exported a large dataset to apply external filters. The e-MEC public interface was queried on 1 July 2025. In the search form, we selected the course type *lato sensu* (*Curso de Especialização*) and entered the Portuguese keyword for health (*saúde*). No temporal filters were applied, ensuring that the query returned the full historical series available in the system. This process produced a spreadsheet file containing 16,179 unique course records.

### 2.2 Data descriptors

Each record contained seven groups of variables. Institutional identifiers included the code assigned to the higher education institution, its full legal name, acronym, and administrative category (such as federal public, state public, municipal public, or private). Program identifiers comprised the unique course-specialization code, course name, and the disciplinary area to which the program is linked according to the Ministry of Education classification. Delivery characteristics specified whether the course was offered via distance learning or in person and listed the approved workload in hours. Geographic attributes included the two-letter abbreviation of the state identifier (UF) in which the course was authorized to operate; this did not apply to distance learning courses. Enrollment capacity and uptake measures recorded the number of authorized student positions and the cumulative number of graduates reported to date. Governance information included the name of the course coordinator and the course's current status (active or inactive). The reported start date of the course was used to establish chronology.

### 2.3 Pre-processing and eligibility

The raw spreadsheet file was imported into Pyspread 2.4 for data cleaning. All character fields were normalized to UTF-8, and obvious misspellings in the headers were corrected programmatically. To focus on programs oriented toward organizational leadership rather than clinical practice, a keyword filter was applied to the course denomination variable. Only rows with titles containing the Portuguese equivalents of *management* or *administration* in any position (case-insensitive) were retained. This filtering reduced the dataset to 3,898 course registrations, which then underwent manual validation. During this process, duplicate entries were identified, and courses whose titles matched the keyword filter but targeted domains outside of health services management (such as health sciences, mental health administration, and QSHE in the oil industry) were excluded. To address misspelled date entries, correct dates were inferred by cross-referencing course codes with their corresponding registration histories from previous and subsequent records. In state identification (UF) fields containing placeholder codes, these codes were first removed. If this was the sole UF value for a record, the entry was excluded from the dataset. Ultimately, this curation process eliminated 645 records, resulting in a final dataset of 3,253.

## 3 Descriptive analysis

We conducted an introductory and descriptive analysis of the main structural and curricular attributes that can be observed in the cleaned dataset of 3,253 *lato sensu* specialization and certification courses related to health services management, active in the Brazilian registry as of 1 July 2025. The resulting dataset is publicly available at doi.org/10.5281/zenodo.16876909. This section emphasizes descriptive reporting, presenting observable patterns in the data without interpreting underlying causes or evaluating program quality.

### 3.1 General characteristics

**Figure 1:**
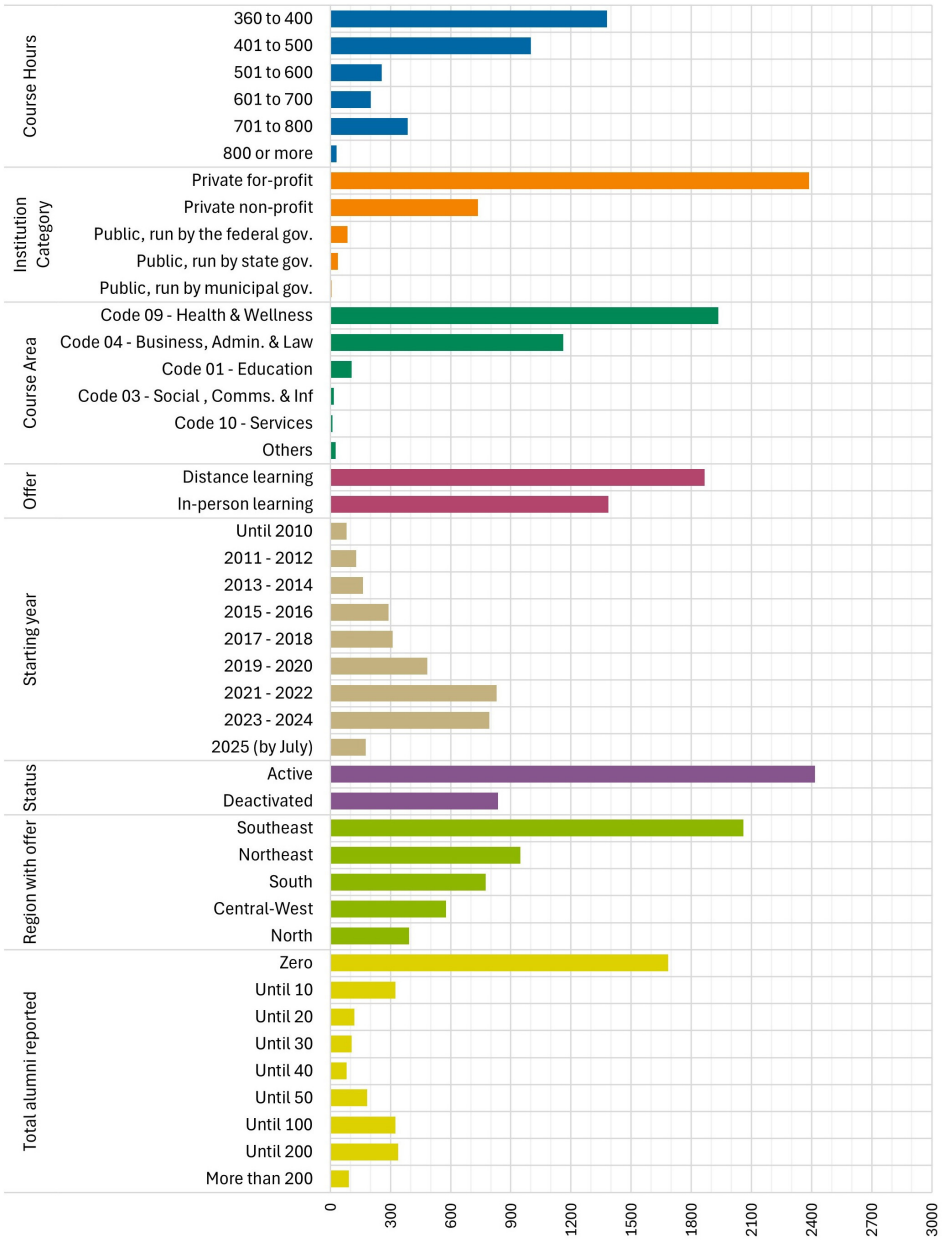
Summary of characteristics of the dataset with the specialization and certification courses offerings related to health services management in Brazil.

Figure 1 presents the most significant institutional and geographic variables. Distance learning is the predominant course delivery modality, enabling courses to reach students across broad geographic areas, often extending beyond the state of the coordinating campus. This expanded catchment area increases enrollment potential in sparsely populated regions while maintaining fixed operational costs.

Despite the presence of courses in all five macro regions, the market remains highly concentrated. A small group of private providers based in the Southeast accounts for the majority of enrollments, with their online courses contributing to totals in multiple states. These institutions effectively export managerial education from established academic centers to states lacking comparable local offerings.

The composition of providers reinforces this pattern. Public universities and nonprofit institutions account for approximately one-third of the programs. Private institutions, many affiliated with larger corporate groups, supply the remaining two-thirds and offer the widest variety of thematic tracks and class sizes.

While e-MEC has functioned as the official registry since 2007, the number of annual course approvals increased notably in 2020 and 2021. This growth coincided with regulatory initiatives promoting remote postgraduate education during the pandemic. More than half of the courses in the current database were launched in 2020 or later, confirming the digital expansion of Brazil's tertiary education sector during this period.

These findings do not suggest that Brazilian healthcare managers are required to hold these certificates, nor do they preclude professionals from obtaining alternative credentials. However, mapping these programs clarifies the competencies emphasized by institutions, the geographic distribution of training capacity, and the curricular areas currently prioritized by the market. These insights establish a foundation for future research examining educational pathways to better understand the knowledge base of Brazil's health service workforce.

### 3.2 Curriculum emphases

**Figure 2:**
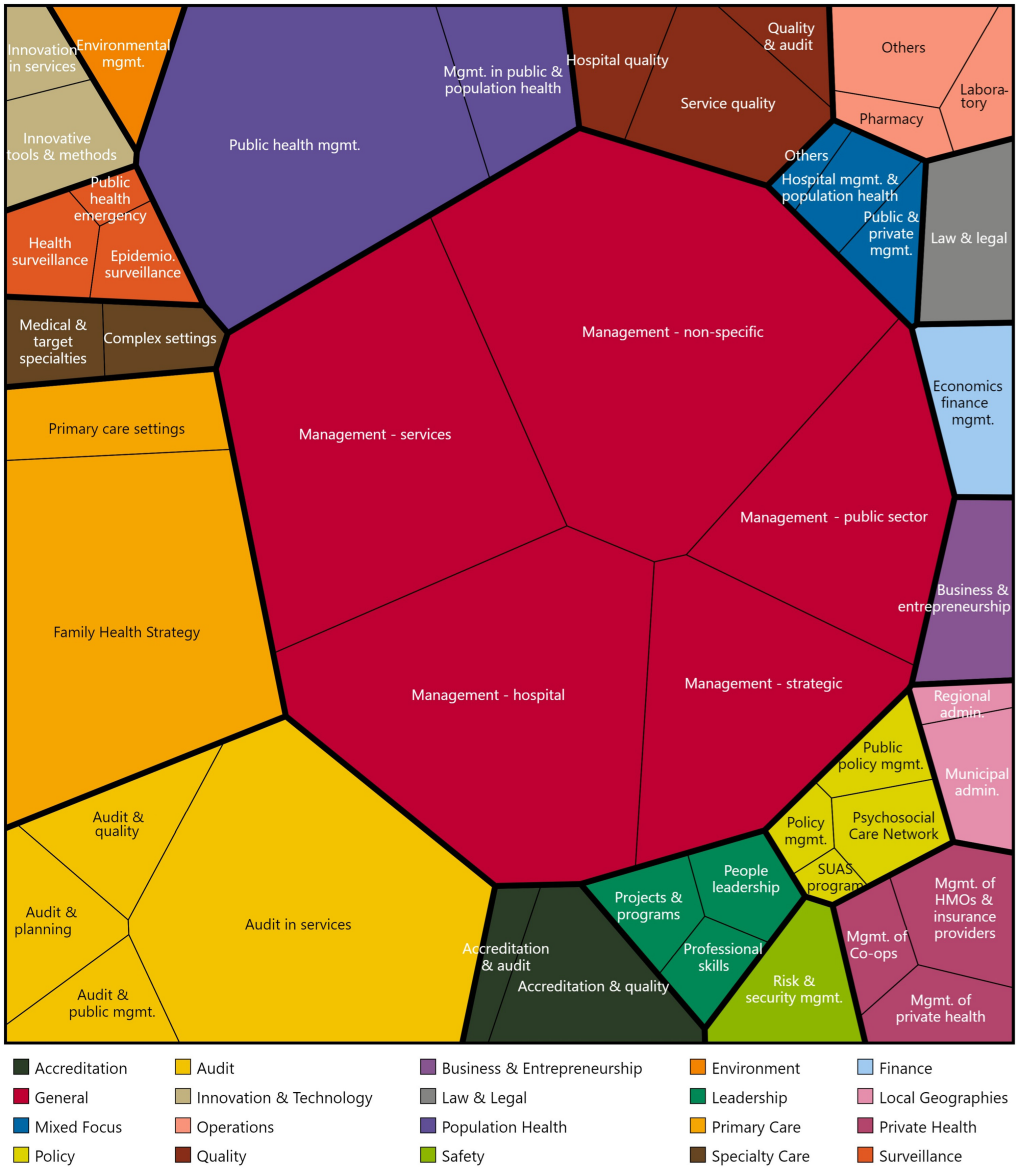
A Voronoi treemap summarizing the curriculum emphases by occurrence of specialization and certification courses offerings related to health services management in Brazil.

Although the dataset categorizes courses, it does not initially focus specifically on management. To address this, a qualitative classification using inductive coding was performed (26). Course titles were recursively evaluated to develop a codebook comprising 20 categories. To illustrate the codebook and its occurrences, a Voronoi treemap was created (27) using the amCharts 5 framework and Kelly's qualitative color palette (28). Figure 2 summarizes the managerial focus of curricula, with the treemap displaying each thematic cluster as a cell sized according to its frequency in the national catalog.

The central area of the treemap features broad polygons representing general courses, reflecting sustained demand for general preparation. Surrounding these are sizable cells dedicated to hospital, strategic, and public-sector management, indicating that managerial education continues to emphasize comprehensive leadership skills. Programs have evolved from general health management to more specialized areas, such as quality assurance, emergency management, and health technology. This progression is illustrated by distinct brown and yellow clusters for audit, service quality, and accreditation, as well as teal cells highlighting people-focused leadership competencies. The treemap also displays medium-sized regions for population health, primary care strategies, and policy governance, suggesting a balanced but not predominant public health emphasis.

Smaller, vividly colored cells represent emerging or niche areas of interest. Finance, law, entrepreneurship, and private health administration are depicted as narrow strips along the perimeter, while even finer segments correspond to environmental management, innovative tools, surveillance, and local geographies. This detailed segmentation indicates limited capacity in these areas despite increasing relevance. The uneven distribution reflects differences in market dynamics, institutional missions, and regional capacities. The diagram highlights areas of market saturation, diversification, and potential investment needs to address competency gaps.

### 3.3 Implications and strategic directions

Because the file documents course offerings rather than graduate characteristics, the data cannot be interpreted as an inventory of practicing managers. Instead, the dataset serves as a proxy for the most visible and accessible training pathways for health service professionals nationwide. A pronounced spatial imbalance is evident: institutions located in the Southeast continue to dominate supply, reflecting the concentration of undergraduate medical schools previously reported in the literature (29). Furthermore, between 2020 and 2024, there was a marked increase in new course offerings and a diversification into technology-oriented and highly specialized subjects. This trend suggests that providers are responding to increasingly segmented and digitally mediated demand.

Approximately eight years ago, Cunha and Hortale (30) reported similar characteristics, with some distinctions. Their study identified the predominance of private sector providers, geographic concentration of institutions, and the centrality of hospital and general management in course portfolios. These features persist, indicating continuity in the field. However, since 2017, distance learning has expanded significantly, the total number of offerings has increased rapidly, and public and philanthropic participation has grown. The curriculum has also diversified, incorporating quality assurance, emergency management, health technology, and other specialized tracks. These developments warrant further analysis using the current dataset.

This dataset offers several practical applications. Regulators may use it to assess the alignment of curricular offerings with strategic priorities, such as primary care management or value-based purchasing. Accreditation bodies can employ the descriptive statistics as a baseline for targeted quality assurance audits. From a research perspective, the institutional and geographic parameters enable potential linkages with labor market or health outcome databases, facilitating analyses of correlations between training density, managerial vacancies, service performance, or regional quality indicators. By assigning each course to one or more of the 20 thematic categories, the codebook enables targeted filtering and aggregation of offerings. For instance, researchers can isolate only courses focused on primary care strategies or health technology to maximize the utility of the dataset for other investigations. Combined with the dataset, the descriptors provided here establish a reference point for future evaluations of Brazil's health management education landscape.

## Data Availability

The datasets presented in this study can be found in online repositories. The names of the repository/repositories and accession number(s) can be found below: https://doi.org/10.5281/zenodo.16876909.
